# Nicotine Suppresses Phagocytic Ability of Macrophages by Regulating the miR-296-3p–SIRP*α* Axis

**DOI:** 10.1155/2023/6306358

**Published:** 2023-02-15

**Authors:** Zhen Liu, Fang Wang, Xiaowu Huang, Zhi Chen, Yicheng Zhao, Yawei Wang, Xiaobo Luo, Guanren Zhao

**Affiliations:** ^1^Medical Supplies Center of Chinese PLA General Hospital, Beijing 100853, China; ^2^Eighth Medical Center of Chinese PLA General Hospital, Beijing 100091, China; ^3^Fourth Medical Center of Chinese PLA General Hospital, Beijing 100048, China

## Abstract

Phagocytic ability of macrophage is responsible for tuberculosis infection. Nicotine has been shown to attenuate the phagocytic ability of macrophage; however, the underlying mechanism remains unclear. Here, we demonstrated that nicotine increased the message RNA (mRNA) and protein expression of signal regulatory protein alpha (SIRP*α*) and enhanced the stability of SIRP*α* mRNA in macrophage. Nicotine decreased the expression of microRNA (miR)-296-3p, which directly targeted the 3′-untranslated region (3′-UTR) of SIRP*α* mRNA in macrophage. Furthermore, nicotine inhibited the phagocytic ability of macrophage by regulating the miR-296-3p–SIRP*α* axis. Moreover, nicotine decreased miR-296-3p expression via increasing c-Myc expression in macrophage. Together, we found that nicotine attenuate the phagocytic ability of macrophage by regulating the c-Myc-miR-296-3p–SIRP*α* signal.

## 1. Introduction

Smoking is a major cause of a highly prevalent airway disease, which results in noxious injury to the lungs and leads to attenuated immunity and increases the susceptibility of human to tuberculosis [1, 2]. Tuberculosis patients with smoking have poor prognosis in China [3]. Macrophage is an important innate immune cell, which is the first line of defense against tuberculosis. After tuberculosis is swallowed, the macrophage can (1) release hydrolytic enzymes to kill tuberculosis; (2) produce interleukins, such as interleukin-1, interleukin-6, interleukin-10, tumour necrosis factor alpha, and interferon gamma; (3) uptake, process, and present effective antigens to T lymphocytes and sensitize T cells to eliminate tuberculosis; and (4) trigger programmed death of themselves, such as apoptosis, autophagy, and necroptosis, to kill tuberculosis [4]. Increasing experimental evidence has indicated that nicotine, an important component in cigarettes, attenuates phagocytic ability of macrophage [5], but the underlying mechanism remains unclear.

Recent experimental evidence indicated that nicotine increased signal regulatory protein alpha (SIRP*α*) expression in microglia and repressed its phagocytic ability [6]. SIRP*α* belongs to the immunoglobulin (Ig) receptor superfamily and negatively regulates the Toll-like receptor (TLR) signaling pathway [7]. The extracellular domain of SIRP*α* has three highly homologous Ig-like domains and multiple glycation sites, which can bind to a ligand, CD47, or some pathogens; thereby, changing its intracellular structure and further regulating the signaling pathways [8–10]. SIRP*α* is observed to be expressed in macrophages and dentritic cells (DCs), but not in lymph T cells [11], suggesting that SIRP*α* may plays an important roles in macrophages. However, whether SIRP*α* is involved in the nicotine-induced decrease in phagocytic ability of macrophages remains unclear.

In the present study, we found that nicotine attenuated the phagocytic ability of macrophages by increasing SIRP*α* expression. Nicotine increased the stability of SIRP*α* message RNA (mRNA) via downregulating microRNA (miR)-296-3p, which directly targeted SIRP*α* mRNA 3′-untranslated region (3′-UTR). Nicotine decreased miR-296-3p expression via increasing c-Myc expression in macrophages. Our findings indicate that nicotine attenuates the phagocytic ability of macrophages via regulating the c-Myc-miR-296-3p–SIRP*α* signal in macrophages.

## 2. Materials and Methods

### 2.1. Cell Culture

Human promonocytic leukemia U937 cells (ATCC^®^ CRL-1593.2™, Manassas, Virginia, USA) were cultured in RPMI-1640 medium, supplemented with 10% fetal bovine serum (FBS; Gibco, 10270, Carlsbad, California, USA), and 293T cells from the Type Culture Collection of the Chinese Academy of Sciences (Shanghai, China) were cultured in Dulbecco's Modified Eagle Medium (DMEM), supplemented with 10% FBS at 37°C in a 5% CO_2_ incubator. The methods for the differentiation of U937 cells into macrophages as previously described [12]. Briefly, U937 cells were cultured in RPMI-1640 medium without FBS for 5 hours and then incubated in RPMI-1640 medium, supplemented with 10% FBS, and 100 ng/mL phorbol 12-myristate 13-acetate; (Tocris Biotechne, 1201, Shanghai, China), for 48 hours. When approximately 70% of the cells became adherent and appeared macrophage phenotype, phosphate-buffered saline was used to wash the non-adherent cells twice. The U937-derived macrophages were used to perform further assays.

### 2.2. RNA Isolation

Total RNAs were extracted in 1 × 10^6^ cells using a Waals™ RNA Extraction Kit (Waals, Chongqing, China) according to the manufacturer's instructions. 50 *μ*L RNase-free H_2_O was used to solve the total RNA.

### 2.3. Quantitative Reverse Transcription Polymerase Chain Reaction

Reverse transcription (RT) of miRs was performed using a TaqMan™ MicroRNA Reverse Transcription Kit (Thermo Fisher Scientific, Shanghai, China), and RT of mRNA was conducted using a QuantiNova Reverse Transcription Kit (QIAGEN, Dusseldorf, Germany) according to the manufacturer's protocols, respectively. Quantitative RT polymerase chain reaction (qRT-PCR) experiments of miRs and mRNAs were performed using a TaqMan™ Advanced miRNA cDNA Synthesis kit (Thermo Fisher Scientific) and a QuantiNova Kit (QIAGEN) according to the manufacturer's instructions, respectively. The primer sequences are shown in Table 1.

### 2.4. Western Blotting

Total proteins were extracted from 1 × 10^6^ cells using a RIPA reagent (Thermo Fisher Scientific), subjected to 10% sodium dodecyl sulfate polyacrylamide gel electrophores (SDS–PAGE) gel, and transferred onto polyvinylidene fluoride (PVDF) membranes. Anti-SIRP*α* antibody (Cell Signaling Technology, Danvers, MA, USA) and anti-glyceraldehyde-3-phosphate dehydrogenase (GAPDH) (Cell Signaling Technology) antibody were used to incubate with the PVDF membranes overnight, respectively, according to the manufacturer's protocol. After washing with 1× phosphate buffered saline with tween 20 (PBST), bands on the PVDF membranes were exposed to a SuperSignal™ West Dura Extended Duration Substrate kit (Thermo Fisher Scientific) according to the manufacturers' instructions.

### 2.5. Phagocytic Ability of Macrophages

Fluorescein isothiocyanate (FITC)-labeled zymosan particles (Sigma–Aldrich, St. Louis, MO, USA) were used to detect the phagocytosis of U937-derived macrophages. The detailed methods were described as a previous study [13]. Briefly, cells were incubated with FITC-zymosan for 30 minutes at 37°C, and evaluated by gating on FITC-positive cells on a FACSCalibur (BD Biosciences, San Jose, CA) after two washes with cold Hanks' Balanced Salt Solution (HBSS).

### 2.6. Transfection

The small interfering RNAs (siRNAs) for SIRP*α* (50 nM) or c-Myc (50 nM), vectors (1 *μ*g), miR-296-3p mimics (50 nM), miR-296-3p inhibitors (50 nM), and the corresponding negative controls (NCs, 50 nM; 1 *μ*g) were transfected into cells using Lipofectamine 3000 reagent (Invitrogen, Carlsbad, CA USA) and B&G Transfection reagent (B&G Biotech, Chongqing, China) according to the manufacturer' protocols, respectively. The siRNA sequences are shown in Table 2.

### 2.7. Vector Construction and Luciferase Activity

The sequences of 1381–1440 nucleotides (nt) and 2156–2215 nt in the 3′-UTR of SIRP*α* mRNA were synthesized and inserted into a pMIR-REPORT plasmid (Applied Biosystems, Foster, California). To confirm the binding sites of miR-296-3p to SIRP*α* mRNA, the mutant sequences, which are corresponding to the wild-type sequence, but the seed binding sites were mutant (mutant 1: CAACCCT to TTCGGA and mutant 2: TCTCAAACCCT to ACTCAATGGGT), were also synthesized and inserted into the same plasmid. For the luciferase reporter assays, firefly luciferase reporter plasmid (1 *μ*g), *β*-galactosidase expression vector (1 *μ*g; Applied Biosystems), and miR-296-3p mimics (50 nM) or NC; 50 nM) were transfected into 293T cells with Lipofectamine 3000 (Invitrogen) and B&G Transfection reagent (B&G Biotech), respectively, according to the manufacturer's protocols. The *β*-galactosidase vector served as a transfection control 24 hours after transfection, the cells were lysed and the luciferase activity was analyzed using a luciferase assay kit (Promega, Madison, WI, USA).

### 2.8. RNA Pull-Down Assay

RNA pull-down assays were performed using a Waals™ RNA Pull-Down Kit (Waals). Briefly, the DNA sequence corresponding to the 3′-UTR of SIRP*α* mRNA was synthesized and used to transcript RNAs by using a (Invitrogen). The RNAs were linked to biotin-labeled oligoes, and then perform the RNA pull-down assays according to the manufacturers' protocol. MiR-296-3p expression levels in the complex pulled down by the 3′-UTR were measured using qRT-PCR.

### 2.9. Statistical Analysis

All data were represented as mean ± standard deviation. Student *t*-test was used to analyze the difference between two groups and Analysis of variance (ANOVA) followed by Tukey honestly significant difference (HSD) was used to evaluate the difference among more than two groups. *P*-value less than 0.05 was considered statistically significant. The graphs were constructed using R (version 4.0.5).

## 3. Results

### 3.1. Nicotine Increases SIRP*α* Expression in Macrophages and Suppresses Its Phagocytic Ability

**Figure 1:**
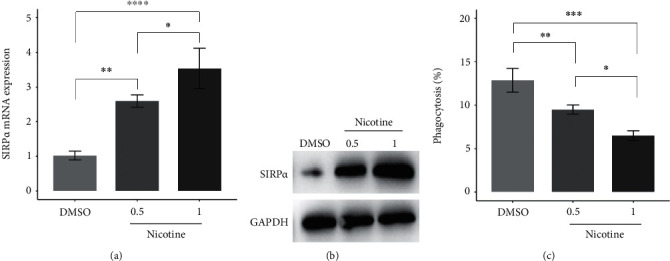
Nicotine increases the levels of SIRP*α* mRNA and protein in macrophages and suppresses its phagocytic ability. (a and b) U937-derived macrophages were treated with nicotine (0.5 and1.0 *μ*M) for 24 hours, DMSO as a negative control. The levels of SIRP*α* mRNA and protein were analyzed by qRT-PCR and western blotting, respectively. GAPDH serves as an internal control. (c) The phagocytic ability of U937-derived macrophages was analyzed in the presence of nicotine. ∗*P* < 0.05, ∗∗*P* < 0.01, ∗∗∗*P* < 0.001, and ∗∗∗∗*P* < 0.0001.

A previous study has demonstrated that nicotine inhibits the phagocytic ability of microglia via increasing SIRP*α* expression [6]. To explore whether nicotine regulates SIRP*α* expression and the phagocytic ability of macrophages, we used nicotine to treat the U937-derived macrophages. qRT-PCR and western blot revealed that nicotine treatment significantly increased the mRNA and protein levels of SIRP*α* in the U937-derived macrophages (Figures 1(a) and 1(b)) and suppressed the phagocytic ability of the U937-derived macrophages (Figure 1(c)). These results suggest that nicotine can elevate SIRP*α* expression and restrict the phagocytic ability of macrophages.

### 3.2. Nicotine Attenuates the Phagocytic Ability of Macrophages by Upregulating SIRP*α* Expression

**Figure 2:**
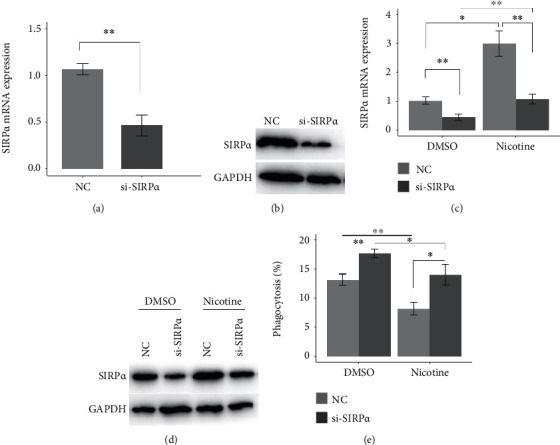
Nicotine attenuates the phagocytic ability of macrophages by increasing SIRP*α* expression. (a and b) 48 hours after U937-derived macrophages were transfected with negative control (NC) or si-SIRP*α*, the levels of SIRP*α* mRNA and protein were analyzed by qRT-PCR and western blotting, respectively. GAPDH serves as an internal control. (c and d) 24 hours after U937-derived macrophages were transfected with NC or si-SIRP*α*, nicotine (1 *μ*M) was used to treat the cells for 24 hours. The levels of SIRP*α* mRNA and protein were analyzed by qRT-PCR and western blotting, respectively. GAPDH serves as an internal control. (e) The phagocytic ability of U937-derived macrophages was analyzed under the same condition. ∗*P* < 0.05 and ∗∗*P* < 0.01.

To evaluate whether nicotine restricts the phagocytic ability of macrophages via upregulating SIRP*α* expression. We used specific siRNA to knock down the SIRP*α* expression, and the results revealed that SIRP*α* siRNAs significantly decreased the mRNA and protein levels of SIRP*α* in the U937-derived macrophages (Figures 2(a) and 2(b)). Furthermore, SIRP*α* siRNAs rescued the nicotine-induced increase in the mRNA and protein levels of SIRP*α* (Figures 2(c) and 2(d)) and rescued the nicotine-mediated decrease in the phagocytic ability of the U937-derived macrophages (Figure 2(e)). These suggest that nicotine represses the phagocytic ability of macrophages by increasing SIRP*α* expression.

### 3.3. miR-296-3p Directly Targets the 3′-UTR of SIRP*α* mRNA and Inhibits SIRP*α* Expression in Macrophages

**Figure 3:**
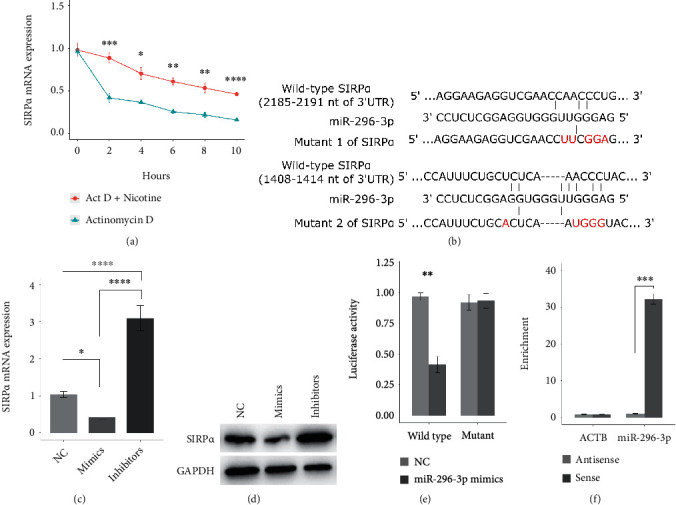
MiR-296-3p directly targets the 3′-UTR of SIRP*α* and inhibits SIRP*α* expression in macrophages. (a) In the presence of actinomycin D (Act D; 2.5 mg/mL) or both Act D (2.5 mg/mL) and nicotine (1 *μ*M) for 0, 2, 4, 6, 8, and 10 hours, respectively. The level of SIRP*α* mRNA was analyzed by qRT-PCR and GAPDH mRNA served as an internal control. (b) The sequences of binding sites of miR-296-3p to SIRP*α* mRNA 3′-UTR and their mutant sequences. (c and d) 48 hours after U937-derived macrophages were transfected with negative control (NC), miR-296-3p mimics, or miR-296-3p inhibitors, the levels of SIRP*α* mRNA and protein were analyzed by qRT-PCR and western blotting, respectively. GAPDH mRNA served as an internal control. (e) 48 hours after 293T cells were transfected, the firefly luciferase activity was analyzed and renal luciferase activity served as a transfection control. (f) Bars show the miR-296-3p or Actin beta (ACTB) levels in the complex pulled down by the sense or antisense of the SIRP*α* mRNA 3′-UTR. ACTB served as a negative control. ∗*P* < 0.05, ∗∗*P* < 0.01, ∗∗∗*P* < 0.001, and ∗∗∗∗*P* < 0.0001.

Next, we sought to investigate the mechanisms by which nicotine increases SIRP*α* expression in macrophages. Given that the mRNA expression of SIRP*α* was regulated by nicotine in the U937-derived macrophages, we considered whether nicotine regulated SIRP*α* mRNA stability or *SIRPα* transcription. We initially investigated the effect of nicotine on the stability of SIRP*α* mRNA by using actinomycin D (Act D, an inhibitor of gene transcription). In the presence of Act D (2.5 mg/mL), nicotine still increased the mRNA levels of SIRP*α* (Figure 3(a)), suggesting that nicotine regulates the stability of SIRP*α* mRNA.

It is well known that miRs can affect the mRNA stability in human cells generally by targeting the 3′-UTR of mRNAs. Thus, we analyzed the potential miRNAs that may bind to SIRP*α* mRNA using the database TargetScanHuamn7.1. We observed 1369 miRs which may target SIRP*α* mRNA (data not shown), and miR-296-3p was considered as the candidate because it not only has a high predictive score, but also has been reported to be negatively regulated by nicotine [14]. The prediction revealed that miR-296-3p may bind to two sites of 1408–1414 nt and 2185–2191 nt in the 3′-UTR of SIRP*α* mRNA which totally contains 3027 nt (Figure 3(b)). We found that miR-296-3p mimics decreased mRNA and protein levels of SIRP*α* in the U937-derived macrophages (Figures 3(c) and 3(d)). Luciferase report assay showed that miR-296-3p mimics inhibited the luciferase activity generated by the luciferase report vector linking wild-type sequences of the two binding sites, but did not influence that generated by the corresponding mutant sequence of the two binding sites in 293T cells (Figure 3(e)). Furthermore, we performed RNA pull-down experiments to determine the enrichment of the SIRP*α* mRNA 3′-UTR with miR-296-3p in the U937-derived macrophage, and the results revealed that miR-296-3p was significantly enriched in the complex pulled down by the SIRP*α* mRNA 3′-UTR (Figure 3(f)). These results suggest that SIRP*α* is a direct target of miR-296-3p in macrophages.

### 3.4. Nicotine Inhibits the Phagocytic Ability of Macrophages via Regulating the miR-296-3p–SIRP*α* Axis

**Figure 4:**
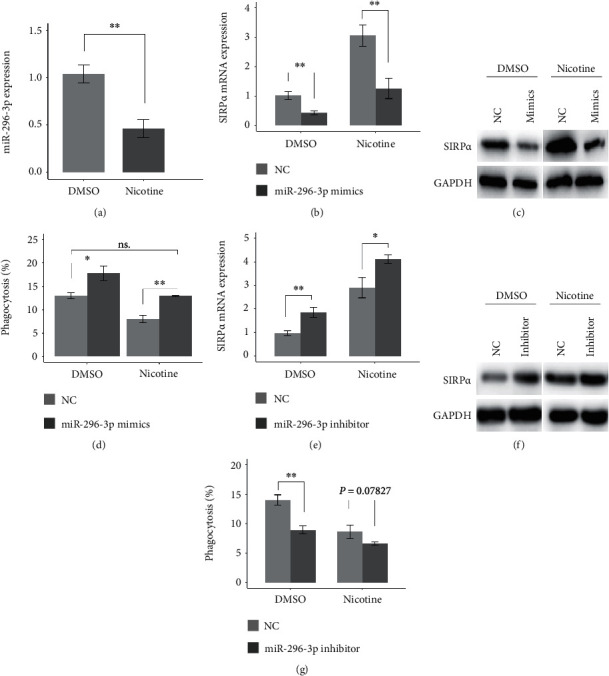
Nicotine inhibits the phagocytic ability of macrophages via regulating the miR-296-3p–SIRP*α* axis. (a) miR-296-3p expression was analyzed by qRT-PCR in U937-derived macrophages treated by DMSO or nicotine for 24 hours. (b and c) 24 hours after U937-derived macrophages were transfected with negative control (NC) or miR-296-3p mimics, nicotine (1 *μ*M) was used to treat the cells for 24 hours. The levels of SIRP*α* mRNA and protein were analyzed by qRT-PCR and western blotting, respectively. GAPDH serves as an internal control. (d) The phagocytic ability of U937-derived macrophages was analyzed. (e and f) Bars and pictures show SIRP*α* mRNA and protein levels in U937-derived macrophages transfected with NC or miR-296-3p inhibitor in the presence of DMSO or nicotine. (g) Bars show the phagocytic ability of U937-derived macrophages transfected with NC or miR-296-3p inhibitor in the presence of DMSO or nicotine. ∗*P* < 0.05 and ∗∗*P* < 0.01.

Next, we demonstrated that nicotine treatment decreased miR-296-3p expression in macrophages (Figure 4(a)). To further determine whether nicotine treatment elevates the mRNA and protein levels of SIRP*α* through downregulating miR-296-3p expression, miR-296-3p mimics were transfected into U937-derived macrophages in the presence of nicotine (1 *μ*M), and the results showed that miR-296-3p mimics rescued the nicotine-mediated increase in the mRNA and protein levels of SIRP*α* (Figures 4(b) and 4(c)). Furthermore, miR-296-3p mimics rescued the nicotine-induced decrease in the phagocytic ability of the U937-derived macrophages (Figure 4(d)). Additionally, miR-296-3p inhibitor significantly increased SIRP*α* mRNA and protein levels in the U937-derived macrophages in the presence of dimethylsulfoxide (DMSO) or nicotine (Figures 4(e) and 4(f)). Moreover, miR-296-3p inhibitor significantly decreased the phagocytosis of the U937-derived macrophages in the presence of DMSO, but not in the presence of nicotine; although, it had a decreased trend (*P* = 0.07827; Figure 4(g)). These results suggest that nicotine inhibits the phagocytic ability of macrophages by regulating the miR-296-3p–SIRP*α* axis.

### 3.5. Nicotine Decreases miR-296-3p Expression by Upregulating c-Myc

**Figure 5:**
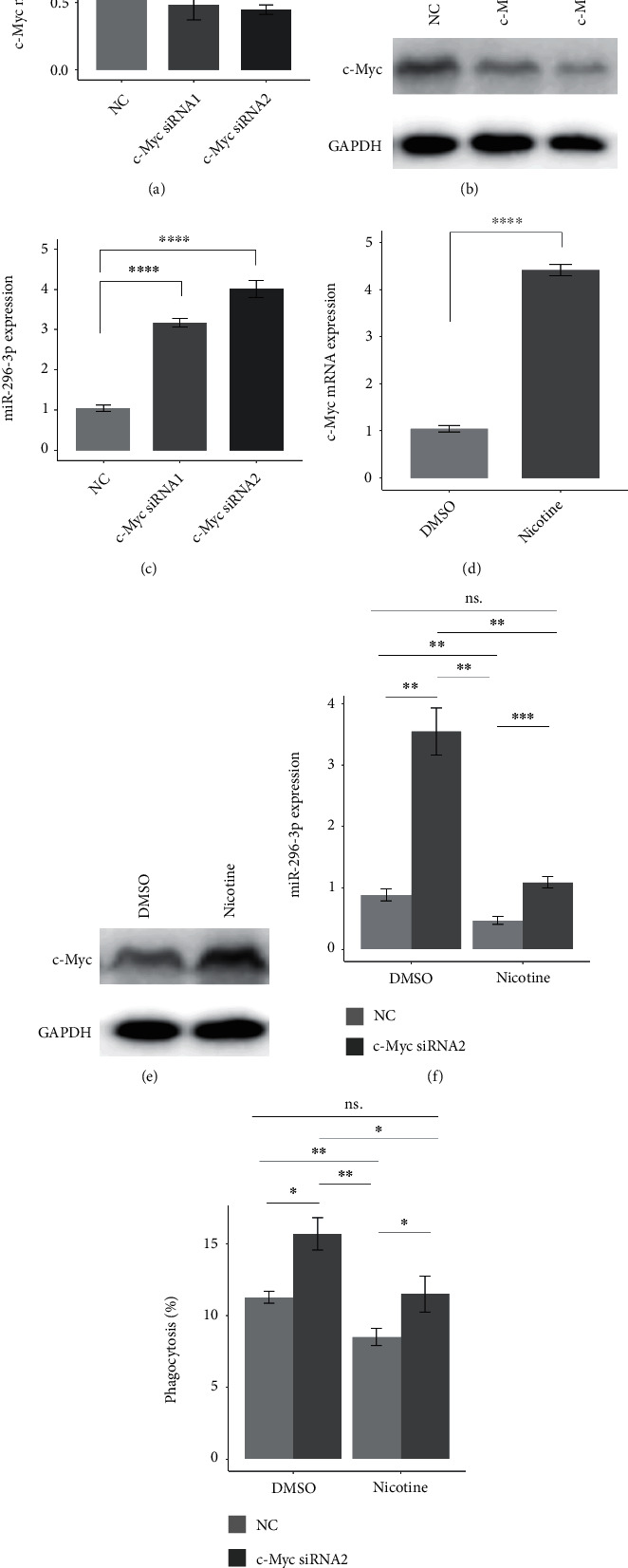
Nicotine decreases miR-296-3p expression by upregulating c-Myc. (a) Bars show c-Myc mRNA expression in U937-derived macrophages transfected with negative control (NC), c-Myc siRNA1, or c-Myc siRNA2, detected by qRT-PCR, and ACTB served as the internal reference. (b) Pictures show c-Myc protein levels in U937-derived macrophages transfected with NC, c-Myc siRNA1, or c-Myc siRNA2, detected by western blot, and GAPDH served as the internal reference. (c) Bars show miR-296-3p expression in U937-derived macrophages transfected with NC, c-Myc siRNA1, or c-Myc siRNA2, detected by qRT-PCR, and ACTB served as the internal reference. (d) Bars show c-Myc mRNA expression in U937-derived macrophages in the presence of DMSO or nicotine, detected by qRT-PCR, and ACTB served as the internal reference. (e) Pictures show c-Myc protein levels in U937-derived macrophages in the presence of DMSO or nicotine, detected by western blot, and GAPDH served as the internal reference. (f) Bars show miR-296-3p expression in U937-derived macrophages transfected with NC or c-Myc siRNA2 in the presence of DMSO or nicotine. (g) Bars show the phagocytosis of U937-derived macrophages transfected with NC or c-Myc isRNA2 in the presence of DMSO or nicotine. ∗*P* < 0.05, ∗∗*P* < 0.01, ∗∗∗*P* < 0.001, and ∗∗∗∗*P* < 0.0001.

Next, we sought to understand the mechanism by which nicotine regulates miR-296-3p expression. A previous study has indicated that nicotine decreased miR-296-3p expression via the PI3K/Akt/c-Myc signaling in nasopharyngeal carcinoma cells [14]. To determine whether this regulatory mechanism also exist in macrophages, we used c-Myc siRNAs to knock down its expression (Figures 5(a) and 5(b)) and found that c-Myc knockdown significantly increased miR-296-3p expression in the U937-derived macrophages (Figure 5(c)). Furthermore, nicotine treatment significantly increased c-Myc mRNA and protein levels in the U937-derived macrophages (Figures 5(d) and 5(e)). Moreover, c-Myc knockdown rescued the nicotine-mediated decrease in miR-296-3p expression in the 937-derived macrophages (Figure 5(f)) and also rescued the nicotine-induced increase in the phagocytosis of the 937-derived macrophages (Figure 5(g)). These results suggest that nicotine inhibits miR-296-3p expression via increasing c-Myc expression in macrophages.

## 4. Discussion

In the present study, we found that nicotine reduced the phagocytic ability of macrophages by enhancing the stability of SIRP*α* mRNA and identified that SIRP*α* was a direct target of miR-296-3p, which could rescue nicotine-induced increased levels of SIRP*α* mRNA and protein as well as nicotine-reduced phagocytic ability of macrophage. Moreover, nicotine decreased miR-296-3p expression by increasing c-Myc expression in macrophages. Therefore, our findings suggest that nicotine inhibits the phagocytic ability of macrophages via the c-Myc-miR-296-3p–SIRP*α* signal.

Macrophage plays important roles in defensing invasion of pathogenic bacteria [15]. The phagocytic ability of macrophages is one of the most important abilities of macrophages in processing pathogenic bacteria [16]. Previous studies have demonstrated that nicotine can reduce the phagocytic ability of macrophages and, thereby, abate the phagocytosis of macrophage to tuberculosis [17], but the underlying mechanism remains unclear. Our findings showed that nicotine attenuated the phagocytic ability of macrophages via increasing the levels of SIRP*α* mRNA and protein in macrophages. SIRP*α* plays a critical role in the establishment of the microenvironment that induces tolerogenic training and its high expression is responsible for the increased infection of pathogenic bacteria [18]. However, the underlying mechanism by which nicotine increases SIRP*α* expression in the presence of nicotine remains to be explored.

Previous studies have reported that SIRP*α* expression is regulated by three miRNAs, including miR-17, miR-20a, and miR-106a in U937 and HL-60 cells [13]. In addition, CD47, a ligand of SIRP*α*, can influence the position and activity of SIRP*α* [19]. In our study, we found that SIRP*α* is a direct target of miR-296-3p, and miR-296-3p can inhibit the levels of SIRP*α* mRNA and protein through binding to two sites in the 3′-UTR of SIRP*α* mRNA in macrophages. Interestingly, miR-296-3p can rescue nicotine-mediated increasing levels of SIRP*α* mRNA and protein, suggesting that miR-296-3p is a major mediator in the regulation of nicotine to SIRP*α* in macrophages. A previous study has revealed that miR-296-3p is negatively regulated by nicotine in nasopharyngeal carcinoma cells [14], suggesting that the regulation of miR-296-3p by nicotine may be general in human cells.

Our study demonstrated that nicotine attenuated the phagocytic ability of macrophages via the miR-296-3p–SIRP*α* axis. We are the first to propose the important role of the miR-296-3p–SIRP*α* axis in the phagocytic ability of macrophages upon nicotine. Because U937-derived macrophages have been used to study human macrophage widely [20, 21], we believe that the miR-296-3p–SIRP*α* axis may exist in human macrophage cells. Furthermore, we demonstrated that nicotine repressed miR-296-3p expression by increasing c-Myc expression. This mechanism has been reported in previous studies in nasopharyngeal carcinoma cells [14]. Previous studies have also indicated that nicotine increased c-Myc expression in human cells [22, 23]. However, the mechanism by which nicotine increased c-Myc expression in macrophages need to be explored in the future.

In conclusion, our findings uncover the molecular mechanism by which nicotine attenuates the phagocytic ability of macrophages by regulating the c-Myc-miR-296-3p–SIRP*α* signal and suggest that the signal may be a potential therapeutic target against pathogen infection.

## Figures and Tables

**Table 1:** Sequences of primers.

Genes	Primers	Sequences
SIRP*α*	Sense	5′-GAGCGTGTCCTACAGCATCCA-3′
	Antisense	5′-AGTGTTCTCAGCGGCGGTATT-3′
GAPDH	Sense	5′-GACCACAGTCCATGCCATCAC-3′
	Antisense	5′-ACGCCTGCTTCACCACCTT-3′
ACTB	Sense	5′-TGTCCACCTTCCAGCAGATGT-3′
	Antisense	5′-TGTCACCTTCACCGTTCCAGTT-3′
c-Myc	Sense	5′-AATGTCAAGAGGCGAACACAC-3′
	Antisense	5′ATTGTTTTCCAACTCCGGGAT-3′

**Table 2:** Sequences of siRNAs.

Genes	siRNAs	Sequences
SIRP*α*	siRNA sense	5′-CAAGCAUUGAGACAGGCAAUU-3′
	siRNA antisense	5′-UUGCCUGUCUCAAUGCUUGUU-3′
c-Myc	siRNA1 sense	5′-GGAAACGACGAGAACAGUUUU-3′
	siRNA1 antisense	5′-AACCGCUUGUGUGUUGCUGUU-3′
c-Myc	siRNA2 sense	5′-AACCUUUGCUGCUCUUGUCAA-3′
	siRNA2 antisense	5′-TGTCACCTTCACCGTTCCAGTT-3′

## Data Availability

The data used to support the findings of this study are included within the article.
